# Applications of generative artificial intelligence in outcome prediction in intensive care medicine—a scoping review

**DOI:** 10.3389/fdgth.2025.1633458

**Published:** 2025-08-05

**Authors:** Tanja Stamm, Mohamed Bader-El-Den, James McNicholas, Jim Briggs, Peng Zhao

**Affiliations:** ^1^ Institute of Outcomes Research, Centre for Medical Data Science, Medical University of Vienna, Vienna, Austria; ^2^ Portsmouth AI and Data Science Centre, Faculty of Technology, University of Portsmouth, Portsmouth, United Kingdom; ^3^ Department of Critical Care, Queen Alexandra Hospital, Portsmouth, United Kingdom

**Keywords:** large language model, generative adversarial network, critical care, survival, mortality, comorbidity

## Abstract

When a patient survives the first 24 h in intensive care, outcome prediction is crucial for further treatment decisions. As recent advances have shown that Artificial Intelligence (AI) outperforms clinicians in prognostication, and especially generative AI has developed rapidly in the past ten years, this scoping review aimed to explore the use of generative AI models for outcome prediction in intensive care medicine. Of the 481 records found in the search, 119 studies were subjected to abstract screening and, when necessary, full-text review for eligibility assessment. Twenty-two studies and two review articles were finally included. The studies were categorized into three prototypical use cases for generative AI in outcome prediction in intensive care: (i) data augmentation, (ii) feature generation from unstructured data, and (iii) prediction by the generative model. In the first two use cases, the generative models worked together with downstream predictive models. In the third use case, the generative models made the predictions themselves. The studies within data augmentation either fell into the area of compensation for class imbalances by producing additional synthetic cases or imputation of missing values. Overall, Generative Adversarial Network (GAN) was the most frequently used technology (8/22 studies; 36%), followed by Generative Pretrained Transformer (GPT) (7/22 studies; 32%). All publications except one were from the last four years. This review shows that generative AI has immense potential in the future, and continuous monitoring of new technologies is necessary to ensure that patients receive the best possible care.

## 1 Introduction

The first medical decision in critical care is whether or not to admit a patient to the Intensive Care Unit (ICU). If this decision is positive and the patient survives the first 24 h, outcome prediction is essential for future treatment over the next few days (1). At this stage, the outcomes of interest include not only short- and long-term survival in and out of the hospital, but also organ or body functioning, the occurrence of new comorbidities and symptoms, quality of life, and the ability to master everyday activities after hospital discharge (2). The latter is vital for patients and their caregivers.

Until now, mainly clinical scores were used to estimate the probabilities of certain outcomes in intensive care. However, in recent years, several studies have shown that prognostications based on Artificial Intelligence (AI) deliver better results than clinical scores (3–5).

Previous AI studies used primarily predictive models to forecast outcomes. However, even state-of-the-art predictive AI methods, such as gradient-boosted trees, are still far from perfect outcome prediction in intensive care (6). With the increasing development of generative models and the more extensive availability of the necessary computing power and large enough datasets, the question arises as to whether generative models or a combination of predictive and generative methods could significantly improve prognostication in intensive care medicine (7). Generative AI comprises data synthesis models, such as Generative Adversarial Networks (GANs) (8), Autoencoders, Variational Autoencoders (VAEs) (9), diffusion models (10), and transformer-based Large Language Models (LLMs) (11).

Two recent reviews on generative AI and outcome prediction in intensive care were published. However, one limited its scope to LLMs (12) and the other focused specifically on critical care nursing (13). No review described the general tasks and applications of generative AI in predicting outcomes in intensive care medicine. Therefore, this was defined as the objective of the present study.

## 2 Methods

### 2.1 Study design and search strategy

A scoping review (14) was conducted due to the exploratory nature of this study and our aim to provide an overview of the diverse use of generative AI in predicting outcomes in ICU, rather than synthesizing all relevant empirical evidence to answer a specific research question, which would be better suited to a systematic review.

**Figure 1:**
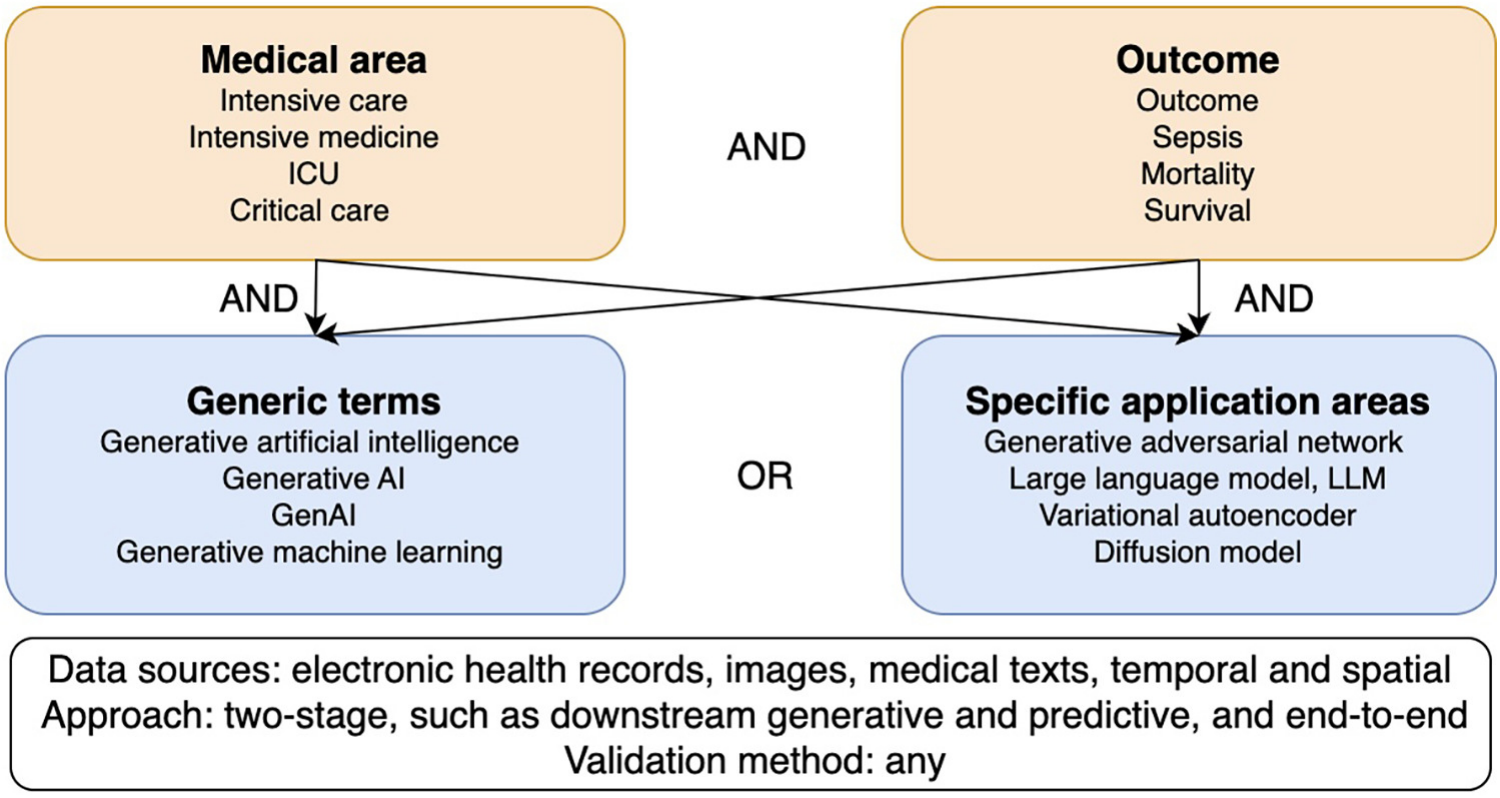
Two-level keyword tree and research field classification diagram.

Our goal was to provide information on the use of generative models to predict outcomes in intensive care. The search strategy was based on a two-level keyword tree (15) covering the areas of intensive care, outcome prediction, generative AI in general, and specific generative AI applications (Figure 1). The search strings were adapted to the respective databases and ran between February 28 and March 17, 2025, in IEEE Xplore, PubMed, CINAHL, Google Scholar, and arXiv (Supplementary Table S1). No limit for publication date was set, and only articles in English were included. We did not limit our scoping review to any level of technical progression. We also included studies dealing with unimodal and multimodal data, time series analyses, as well as studies that used images or medical text. Genetic studies were excluded due to the rare availability of these data in electronic health records. Although outcome predictions in intensive care came most often from tabular data, we did not exclude studies that used images or text. Furthermore, all study designs were included, except for commentaries and opinion letters.

### 2.2 Study selection

Duplicates were removed. The first author (TS) screened the titles and abstracts for eligibility. Full-text records were consulted when necessary. A second automated abstract screening was conducted for quality control using ChatGPT’s freely accessible GPT-3.5 model. The prompt used for this computerized eligibility assessment was as follows: “*You are a critical researcher. Below is an abstract of a study. Please tell me if you would include this in a literature review. This review aims to describe the applications of generative artificial intelligence in outcome prediction in critical care. Only studies that used generative AI models and focused on outcome prediction in critical care must be included*.” In case of disagreement between the human and machine assessments, ChatGPT’s arguments were discussed in a study team meeting, and a joint decision was made. Human judgment was prioritized.

### 2.3 Data extraction and reporting

Full texts of suitable publications were obtained. The publication year, the name of the journal or conference, the aim and outcome domains of the research, the dataset, and the generative models were extracted from the full text records using a custom data extraction sheet. There was no overlap across all three use cases in any one study. We used the Prediction model Risk Of Bias ASsessment Tool (PROBAST) +AI (16) to assess the quality of the studies in terms of the selection of participants and data sources, predictors, outcomes and analysis. The Preferred Reporting Items for Systematic Reviews and Meta-Analyses (PRISMA) guidelines (17) were followed when presenting the results.

## 3 Results

**Figure 2:**
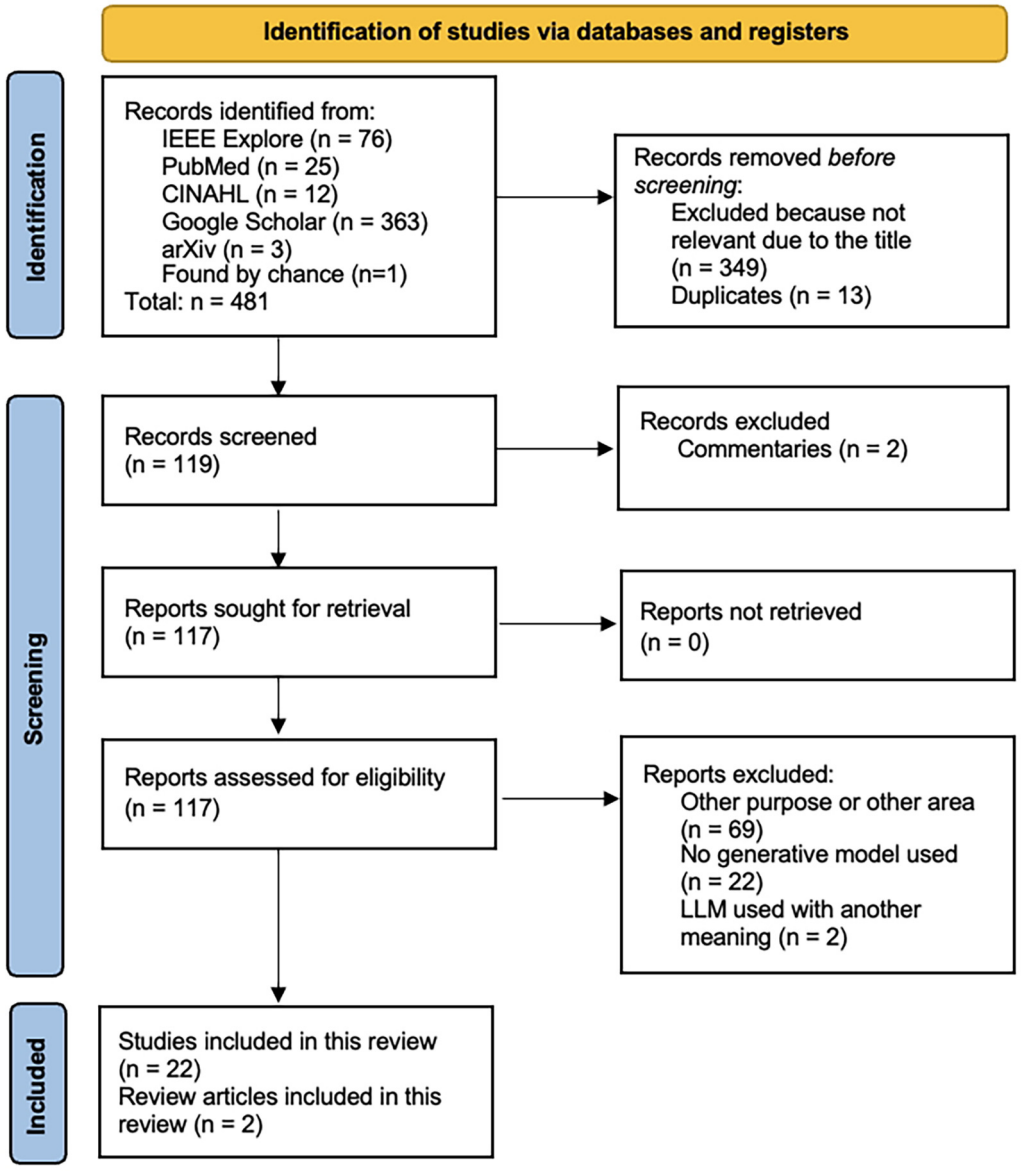
PRISMA Flow Chart for study selection and assessment.

Of the 481 records found in the search, 119 studies were subjected to abstract screening and, where necessary, also full-text review. Twenty-two studies and two review articles were finally included in the present scoping review (Figure 2; Tables 1, 2). The papers excluded during abstract screening and full text review are shown in Supplementary Table S2.

**Table 1:** Details of the 21 studies included in this review.

#	Authors	Year	Journal or conference	Aim	Dataset	Generative model
1	Wei et al. (19)	2021	IEEE Access	In-ICU mortality prediction	MIMIC-IV	GAN
2	Yang et al. (20)	2023	Heliyon	In-ICU mortality prediction	MIMIC-III	Variant of GAN (c-med GAN)
3	Shariat et al. (21)	2024	International Conference on Web Research (ICWR)	Prediction of neonatal infections	113,378 neonates admitted in the year 2022	GAN
4	Wang et al. (22)	2024	Inter. Conf. on Biomed. Engineering and Applications (ICBEA)	Acute pain prediction	UNBC-McMaster shoulder pain dataset	GAN
5	Ravikumar et al. (23)	2024	IEEE Access	Skin infection prediction	HAM10000, ISIC 2018 challenget	GAN
6	Ryan et al. (24)	2013	Biomedical Sciences and Engineering Conference (BSEC)	In-ICU mortality prediction	Physionet/CinC 2012 Challenge data	Deep Boltzmann machine
7	Apalak and Kiasaleh (25)	2022	IEEE Access	Sepsis prediction	2019 PhysioNet Computing in Cardiology Challenge dataset	Recurrent conditional GAN
8	Kim et al. (26)	2020	Intern. Conf. on Pattern Recognition (ICPR)	In-ICU mortality prediction	Physionet Challenge 2012 – 4,000 ICU stays with 80.5% missings)	GAN
9	Zhang et al. (18)	2023	International Conference on Tools with Artificial Intelligence (ICTAI)	In-ICU mortality prediction	MIMIC-III	MedCT-BERT
10	Mesinovic et al. (27)	2024	Journal of the American Medical Informatics Association	Survival analysis	MIMIC-IV	Conditional VAE
11	Ramos et al. (28)	2021	Ann. Inter. Conf. of the IEEE Engin. in Med. and Bio. Soc. (EMBC)	Sepsis prediction	MIMIC-III	VAE
12	Rao et al. (29)	2024	IEEE Int. Conf. on Industry 4.0, AI and Comm. Tech. (IAICT)	Anomaly detection	MIMIC-III/IV	GAN
13	Vurgun et al. (30)	2024	Advan. in Med. Found. Mod.: Explainab., Robustn., Secur., a. B.	Cardiac arrest identification	Data from the Hospital of the University of Pennsylvania	GPT-4 and 51 open-source LLMs
14	Pathak et al. (31)	2024	IEEE Journal of Biomedical and Health Informatics	Acute respiratory distress syndrome identification	Data from two hospital in Altanta	NLP Pipeline with BERT model
15	Madden et al. (32)	2023	Intensive care medicine	Creation of patient summaries	Two sets of medical notes	GPT-4
16	Lin et al. (33)	2025	Journal of the American Medical Informatics Association	In-ICU mortality prediction	MIMIC-IV	BERT
17	Pabon et al. (34)	2024	European Journal of Heart Failure	Feature extraction	6,263 patients enrolled in the DELIVER trial	GPT-3.5
18	Parizad et al. (35)	2024	Intern. Conf. on Soft Comput. and Mach. Intell. (ISCMI)	Prediction of hospital readmission	MIMIC-III	ChatGPT
19	Chung et al. (36)	2024	JAMA surgery	Prediction of perioperative risks and prognosis	Retrosp. collected data from electronic health records	GPT-4 Turbo
20	Amacher et al. (37)	2024	Resuscitation Plus	Prediction of outcomes after cardiac arrest	Data of Swiss cardiac arrest patients admitted to ICU	ChatGPT-4
21	Yoon et al. (38)	2025	Journal of the American Medical Informatics Association	Prediction of 30-day out-of-hospital mortality	MIMIC-IV	GPT-4
22	Contreras et al. (39)	2024	arXiv	Prediction of delirium in the ICU	Three ICU datasets: eICU, MIMIC, and UFH	New LLM-based model (DeLLiriuM)

**Table 2:** Details of the 2 review papers included in this review.

#	Authors	Year	Journal or conference	Scope	Number of papers included	Design
1	Shi et al. (12)	2024	arXiv	LLMs in critical care	24	Scoping review
2	Porcellato et al. (13)	2025	Nursing reports	Generative AI in critical care nursing	24	Systematic review

**Figure 3:**
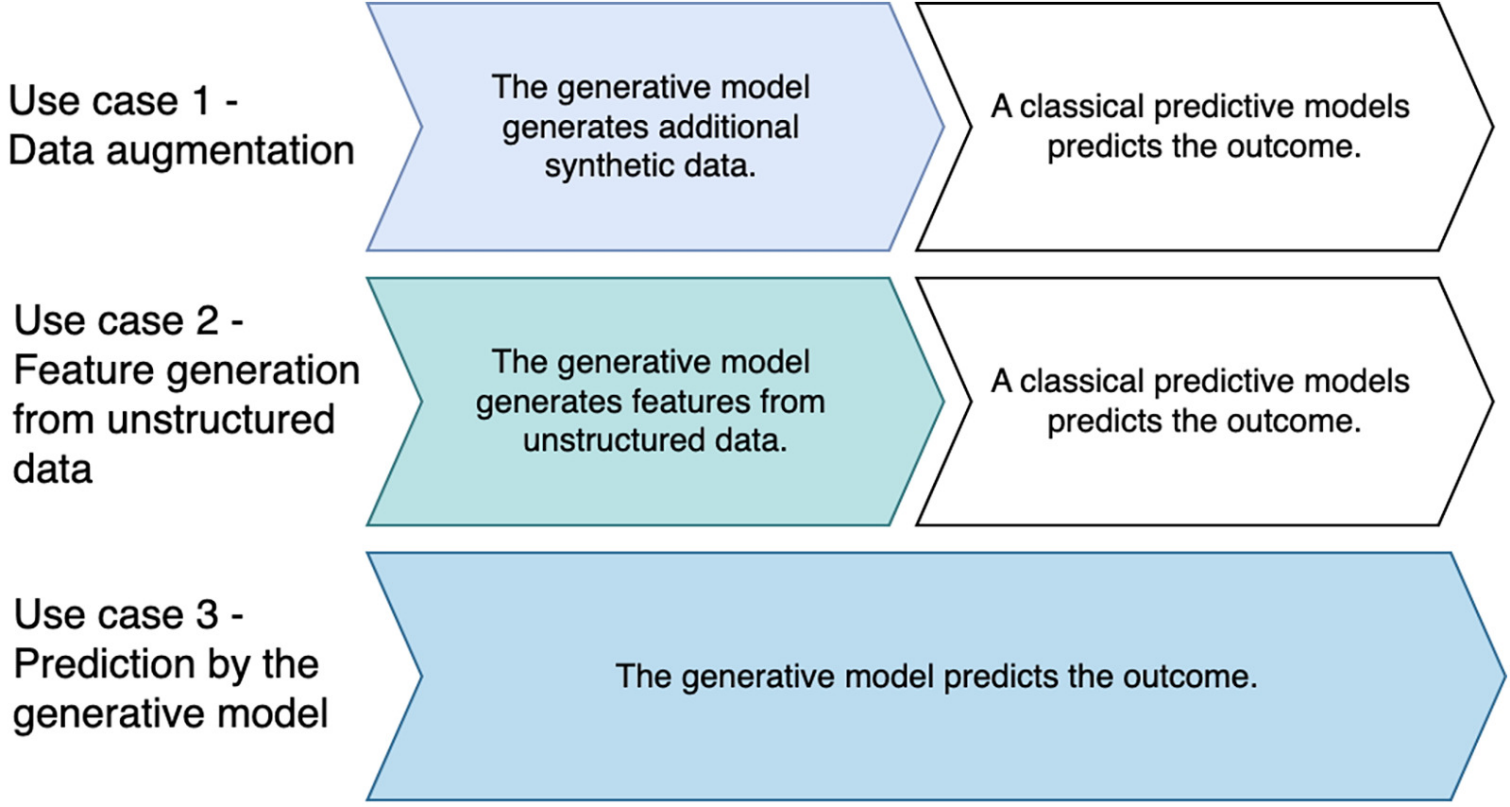
Prototypical use cases for the use of generative AI in outcome prediction in intensive care medicine.

The studies were categorized into three prototypical use cases for the use of generative AI models in the prediction of outcomes in intensive care medicine: (i) data augmentation, (ii) feature generation from unstructured data, and (iii) prediction by the generative model. In the first two use cases, the generative models worked together with downstream predictive models that performed the prediction. In the third use case, the generative models made the predictions themselves (Figure 3). While studies predominantly fitted one use case, some integrated multiple generative approaches [e.g., (18)].

### 3.1 Use case 1 – data augmentation

As expected, generative AI was used to compensate for class imbalances by producing additional synthetic data to improve the performance of downstream classical predictive models. Two studies (19, 20) focused on in-ICU mortality prediction and used GAN to supplement data in the minority class. In the first study, Wei et al. (19) applied GAN for tabular data augmentation to improve the performance of a downstream extreme gradient boost tree (XGBoost), leading to an Area Under the Receiver Operating Curve (AUROC) of greater than 0.90. Additional synthetic cases were needed in this study because the number of deceased patients in the original dataset was considerably lower than that of survivors. Moreover, a classifier layer was added to the GAN that reduced falsely non-binary GAN-generated values to binary ones. The missing values were imputed by filling in mean values or the majority subtype for the categorical features. Finally, age and blood urea nitrogen (BUN) contributed the most to the XGBoost model, shown in a Shapley additive explanation (SHAP) analysis. From a medical perspective, the results are meaningful, as age and kidney function play an important role in survival. In the second study on mortality prediction in critical care, Yang et al. (20) developed and evaluated the conditional medical GAN (c-med GAN) to improve the synthesis of discrete parameters consisting of an additional autoencoder that generated the new data separately from their labels and a functionality that added the class labels as constraints. The autoencoder network was used because GAN generally had difficulties in synthesizing discrete data that are often included in electronic health records to a satisfactory quality. Similar to the first study, the AUROC of the model that included the c-med GAN was >0.90. Shariat et al. (21) used imbalanced tabular data to predict neonatal infections and applied a simple GAN to synthesize additional data in the minority class to improve the performance of downstream predictive models, including Random Forest (RF), Support Vector Machine (SVM), Bagging, and Boosting. RF and Bagging outperformed the other models achieving an F1-score of >0.95.

Two further studies utilized GAN to augment image datasets. In the first study, Wang et al. (22) used synthetic, GAN-created pictures of facial expressions of ventilated ICU patients whose faces were often obscured by intubation or masks to predict acute pain. However, while a tool to evaluate pain would be useful both for patient experience and potential avoidance of some physical complications of critical illness, sedation regimes may include deep muscle relaxation, and thus, facial movement could be attenuated or abolished altogether. In the second study, Ravikumar et al. (23) reviewed approaches to create synthetic images of skin lesions and tested a conditional GAN that, together with a downstream DenseNet-201, outperformed Visual Geometry Group 16 and Support Vector Machine in predicting skin infections, achieving an accuracy of approximately 82%. While this approach could indicate specific symptoms, it would probably contribute little to the prediction of the general state of health of critically ill patients.

Another task of generative models in the area of data augmentation was imputation. A major problem with ICU data is the frequently large proportion of missing measured values. The first of the studies in this area and, at the same time, the oldest of the ones included in this entire review, used a generative model, a deep Boltzmann machine, to learn the distribution of time series data and impute missing values to improve the performance of a downstream neural network to predict in-hospital mortality in ICU patients (24). Apalak and Kiasaleh (25) applied a conditional GAN where the generator and discriminator were Long Short-Term Memory networks (LSTMs) to impute missing tabular time series data. They showed that this approach improved the performance of another downstream predictive LSTM in forecasting sepsis, leading to AUROC values between 0.93 and 0.94. LSTM could be a promising approach, as it can process the sequential medical measurement data. Kim et al. (26) used GAN to impute missing values by applying a slightly different approach. By randomly dropping a certain percentage α of values from an original multivariate time series input dataset X, they constructed a corrupted dataset $\bar{X}$ while retaining the original labels [Equation 1; (26)]:

(1) $$\bar{X}∼\;{Drop}_{\alpha }(\bar{X}/X)$$

$\bar{X}$ was then used as input data instead of X, and the generator replaced the dropped values with the average values in each iteration, thus reducing noise during imputation. Kim et al. (26) showed that the synthesized dataset worked similarly in a downstream predictive model to forecast in-ICU mortality compared to the original data. They measured imputation performance using mean squared error and mean absolute error and achieved the highest values with their new approach of 0.48 and 0.37, respectively. Zhang et al. (18) proposed a multimodal learning model for in-ICU mortality prediction in critical care. They first used a Bidirectional Encoder Representations from Transformers (BERT) model to generate text embeddings of the medical records of each patient. This created missing values due to irregularities in intervals in time series data. Therefore, the authors adapted a GAN to interpolate the time series data and used feature correlations in the original dataset to assess the appropriateness of the interpolated data. This new approach, called MedCT-BERT outperformed several other generative and predictive approaches, achieving an AUROC of 0.89, including BioBERT. The textual embeddings of the data could capture nuances that plain structured data might miss.

Two further studies used VAE to augment tabular data. Mesinovic et al. (27) imputed serial lab value data from the ICU using a conditional VAE and combined this model with a right-censored time-to-event survival analysis instead of just a discrete outcome label. The conditional VAE was used in this study to learn the latent representation of the input dataset. This approach led to a mean AUROC > 0.67. In another study, Ramos et al. (28) first imputed missing data with a forward-filling strategy, then applied VAE to learn the latent representation of the input data and, lastly, used unsupervised clustering to detect rare abnormal events and, thus, predicted septic shocks; this model showed a comparable performance as a supervised LSTM network, achieving an AUROC of 0.82. This study differs from many others because no labeled dataset was used. Overall, it proposed a potentially useful tool to prompt the initiation of preventive measures, such as the prescription of antibiotics.

Rao et al. (29) attempted to filter out abnormal values in physiological parameters that could lead to false alarms during data synthesis. The authors used distance-related anomaly scores calculated at the generator and the discriminator of the GAN, leading to an accuracy of 0.97. The new model outperformed a Convolutional Neural Network-autoencoder-based anomaly detection model. From a medical perspective, this new method could have the potential to detect risks of events other than sepsis and shock onset, for example, sudden cardiac arrest.

### 3.2 Use case 2 – feature generation using unstructured data

In this use case, generative AI was used to create new features based on unstructured textual data for a downstream predictive model. Vurgun et al. (30) tested 51 open-source LLMs against GPT-4.0 to extract in-hospital cardiac arrest events using discharge summaries, progress notes, and tabular data, with several other open-source models demonstrating competitive results, such as Mistral-Nemo-Instruct-2407. The highest AUROCs achieved were between 0.91 and 0.90. However, this was not a prediction of a future event, as the cardiac arrest had already been determined by the care team. Pathak et al. (31) used BERT to classify radiology reports to predict acute respiratory distress syndrome (ARDS). However, while technically novel, applications like ARDS identification from radiology reports may offer limited added value if clinicians already documented these diagnoses. Maden et al. (32) used GPT-4 to create patient summaries from daily free-text medical notes, thereby making this information accessible for critical care decisions and outcome prediction. The study concluded that the clarity of the prompts determined the quality of the summaries. The highest AUROCs were between 0.75 and 0.88. Moreover, writing patient summaries would tie up considerable resources and could therefore be usefully replaced by AI. Lin et al. (33) predicted in-hospital mortality based on radiology reports, chest x-rays, and clinical ICU data. Convolutional neural network processed the image data. The token embeddings from the last layer of a BERT model were used as latent representations of the radiology reports. Feature fusion from all three data sources, clinical data, chest x-rays and radiology reports, led to a slightly better AUROC than only one or two feature sources alone (+0.01). Pabon et al. (34) applied GPT-3.5 to extract information on the left ventricular ejection fraction from medical records. However, tabular data were used in this study for data extraction and not the unstructured text.

Parizad et al. (35) used ChatGPT in a different way than the studies described above. The authors of this study first asked ChatGPT for advice on which features to include in a frailty index, but then extracted the actual data from the clinical notes using non-generative natural language processing techniques.

### 3.3 Use case 3 – prediction by the generative model

Generative models were also used to forecast outcomes in intensive care medicine. GPT-4 Turbo showed an acceptable performance in predicting mortality (F1 = 0.86) and clinical scores of the American Society of Anesthesiologists (F1 = 0.50) using medical notes on the instructions. However, temporal predictions (e.g., ICU stay duration) performed poorly (MAE = 1.1 days) (36). While the two F1 scores were 76% and 194% better than a random classifier at baseline, the mean absolute error of 1.1 days was the same as the dummy regressor baseline. Moreover, a comparison with the actual decision of an intensive care physician would have been desirable here, too. Amacher et al. (37) asked ChatGPT-4 to predict the occurrence of poor neurological outcome and the likelihood of survival of cardiac arrest patients from a Swiss ICU dataset, but used, in contrast to the previous study, the tabular data in the prompts. In Amacher’s study, ChatGPT-4 showed only a similar performance (AUROC = 0.85) as clinical scores derived from health professionals (AUROC = 0.83). Furthermore, Yoon et al. (38) tested LLMs tuned with instructions to predict mortality from discharge notes at 30 days after the patients had left the hospital; in this study, GPT-4 showed the best result (32.2% in F1 metrics compared to 28.9% for best-performing supervised model). Contreras et al. (39) trained a novel LLM-based delirium prediction model using electronic health record data from the first 24 h of ICU admission from three openly accessible databases. The new model used a clinical 345 million-parameter LLM (GatorTronS) as the backbone and performed better (AUROC ranging being 0.77 and 0.82 in two external validation datasets) than three other deep learning models, namely a Neural Network, a Transformer model, and Mamba. The features identified in the SHAP analysis were consistent with the usual accepted risk factors for delirium, and only urine specific gravity was unexpected and new.

Furthermore, the two narrative reviews (12, 13) outlined several clinical areas where LLMs and other AI methods could show their advantages, such as the integration of multimodal and unstructured data, the creation of patient summaries and the prediction and prognostication, the second review specifically highlighting how these applications could support critical care nursing.

### 3.4 Technology used and bibliometric findings

GAN was the most frequently used technology (8/22 studies; 36%), followed by GPT (7/21 studies; 32%). All publications except one were from the last four years; the oldest was published in 2013. Medical Information Mart for Intensive Care (MIMIC) in different versions was the most frequently used dataset (10/22 studies; 45%). No study predicted long-term outcomes in intensive care medicine using generative AI.

### 3.5 Risk of bias assessment

While the predictors, outcome and analysis were described transparently in most studies (Table 3), information regarding the selection of the study participants and datasets was often lacking (22, 27, 31). Moreover, pragmatically defined sample sizes (26) and the use of commercial models on a smaller scale, due to cost constraints, compared to the unrestricted use of freely accessible models (30), could have introduced additional bias.

**Table 3:** Risk of bias assessment according to Prediction model Risk Of Bias ASsessment Tool (PROBAST) +AI (16).

#	Authors	Year	Risk of bias introduced by the			
			Selection of participants and data sources	Predictors or their assessment	Outcome or its determination	Analysis
1	Wei et al. (19)	2021	?	+	+	+
2	Yang et al. (20)	2023	?	+	+	+
3	Shariat et al. (21)	2024	?	+	+	+
4	Wang et al. (22)	2024	−	+	+	+
5	Ravikumar et al. (23)	2024	+	+	+	+
6	Ryan et al. (24)	2013	+	+	+	+
7	Apalak and Kiasaleh (25)	2022	+	+	+	+
8	Kim et al. (26)	2020	?	+	+	+
9	Zhang et al. (18)	2023	?	+	+	+
10	Mesinovic et al. (27)	2024	−	+	+	+
11	Ramos et al. (28)	2021	+	+	+	+
12	Rao et al. (29)	2024	−	+	+	+
13	Vurgun et al. (30)	2024	?	?	+	+
14	Pathak et al. (31)	2024	?	+	+	+
15	Madden et al. (32)	2023	−	+	?	+
16	Lin et al. (33)	2025	+	+	+	+
17	Pabon et al. (34)	2024	+	?	+	?
18	Parizad et al. (35)	2024	?	+	+	+
19	Chung et al. (36)	2024	?	+	+	+
20	Amacher et al. (37)	2024	+	+	+	+
21	Yoon et al. (38)	2025	+	+	+	+
22	Contreras et al. (39)	2024	+	+	+	+
Review 1	Shi et al. (12)	2024	NA	NA	NA	NA
Review 2	Porcellato et al. (13)	2025	NA	NA	NA	NA

“+” refers to a low; “−” to high risk of bias; “?” indicates unclear information.

## 4 Discussion

Although generative models have been used in many areas, primarily for images and text, our results clearly show their value in tabular data from electronic health records in outcome prognostication in intensive care medicine. The recent publication dates of the studies highlight the topicality of this research field and the immense innovation potential in using new technologies for unusual tasks, such as making a generative model take over prediction tasks on its own. However, generative AI research still remains narrowly focused on short-term mortality. Future work should target patient-centered long-term outcomes.

Although some technologies have rarely been used in outcome prediction in intensive care medicine, they could be adapted and applied to this field even more in the future. Examples are Retrieval Augmented Generation (RAG) or diffusion models. A study, for example, using RAG to retrieve information from medical reports, was excluded from this review because it did not focus on predicting outcomes in intensive care medicine (40). Likewise, another study applied a novel combination of a diffusion model with an upstream autoencoder block to forecast time series data on heart rate and blood pressure (41), but did not forecast outcomes in critical care either.

Generative AI further showed that it had the potential to enrich tabular data with additional information from different sources. In the reviewed studies, the source of information was mainly medical notes. Images were used less frequently and only in special contexts, e.g., to predict facial expressions of pain or skin lesions. A special category of studies did not use the generative model to extract features from medical notes, but rather asked the generative model, such as ChatGPT for features to extract from medical notes (35). Similarly, other studies also used ChatGPT to obtain medical advice (42). However, there are controversial views on the quality and evidence-based nature of the recommendations derived from ChatGPT (43, 44). Unrelated work (45) suggested potential options for psychosocial feature extraction; however, the actual feature extraction was performed by experts in this study (45).

An interesting distinction is whether the approaches in the studies are two-stage approaches or end-to-end approaches. All studies that we classified as use cases 1 and 2 contain two-stage approaches per se, as the generative model was used in the first case to supplement incomplete or infrequently available data or to generate new parameters from unstructured data. For this purpose, a generative model was always used first, followed by a predictive model. Only in the third use case, the predictive model made the prediction itself (end-to-end approach).

Denoising referred to irregularly sampled time series values, abnormally imputed values, and simple errors (26). Denoising autoencoders were originally designed to prevent the output sequence of the encoder from being equal to the input data as this would make the autoencoder obsolete. The denoising autoencoder would, therefore, use noisy or corrupted input for the decoding, but calculate the encoder loss based on the original input data.

In some studies, although the data science method was new and innovative, the medical aim was questionable. Especially when diagnoses could be made easily or, for example, a chest X-ray must have been viewed by a specialist anyway, as not only was a diagnosis made by that, but other parameters were also assessed. However, it is still possible to use the experiments to further develop the technology or contribute to the wider availability of medical knowledge beyond that of experts. This could be relevant for the automated creation of summaries, but also for doctors in training or when certain experts are unavailable.

Generative models are computationally resource-intensive. This might create additional data protection issues, and secure processing environments would be needed to analyse patient data. To avoid such problems, the publicly accessible MIMIC dataset was probably the most often used database in the studies included. However, perhaps the results should also be validated in different datasets in the future. In addition, generative models can help solve data protection problems. Completing missing data can be extended to a data synthesis problem, and data synthesised in sufficient quality will be accepted more and more as a replacement of the original data. Since data synthesis is a generic task, methods from other fields can also be applied to predict outcomes based on ICU data. For example, Neves et al. developed a GAN with only two layers and a hyperbolic tangent activation function in the output layer for the imputation of medical data, which could synthesize data faster (46).

The clinical implications of using generative AI in intensive care units extend beyond outcome prediction, which was the scope of this review. At present, we are still a long way from automated treatment. Beyond liability issues, ethical considerations, bias, hallucinations, the lack of necessary qualifications in healthcare professionals, and potential changes to work processes, medicine also involves an element of humanity that AI cannot yet easily replace. However, it has already been shown that machines can not only provide effective decision support in treatment, but also engage in more empathetic dialogues than clinicians when interacting with patients and their relatives (47). Nevertheless, until now, the final decision on treatment has always been made by a human, albeit supported by AI.

Ethical implications of using generative AI in real-time critical care settings, including hallucinations, bias, accountability, and data privacy, as well as regulatory issues should be addressed through future research, which should also take into account multi-stakeholder perspectives. Real-time deployment of generative models [e.g., ChatGPT-4 (37)] requires rigorous hallucination safeguards, especially when tabular data inputs may propagate biases.

Practical implementation of generative AI in intensive care medicine will depend heavily on its effectiveness, regulatory approval, technical interoperability with electronic health records and other systems, the skills of health professionals and hospital policies related to patient pathways and digital medicine. The detailed discussion of these aspects is beyond the scope of this review. Further research should focus on addressing them.

Interpretability and explainability of generative AI are crucial for its acceptance, adoption and deployment in clinical practice (48). Further research should address this area, incorporating the perspectives of clinicians, patients and their caregivers.

A limitation of this review might be its static nature, while the technology is advancing at a high speed. Novel technologies and new application areas might be published during or after this review. Work on the first update of this review is therefore scheduled to begin one year after the publication date. Following these arguments, more studies could also have been expected in this review. However, in a review of scoping reviews, Tricco et al. (48) showed that the average number of studies included in 494 scoping reviews was 118, ranging from 1 to 2800. This is comparable to the 119 records that were assessed in detail in the present study. Moreover, the scoping review on LLMs in intensive care (but not limited to outcome prediction) also included in this review (13) ended with a similar number of 24 articles as our final selection. In addition, as also done in the aforementioned paper, we limited the keyword search to titles and abstracts in some databases, as we were specifically looking for studies that used generative methods in outcome prediction and did not just mention generative AI, for example, in the discussion. A further limitation of our study could be that preprint studies without peer review were also considered. However, we were transparent about this, and the publication origin of the studies is indicated accordingly in Tables 1, 2. Another limitation of our study is that the second abstract screening was conducted using ChatGPT’s 3.5 model. Inclusion of a second human review could have enhanced the methodological rigor of this process. This review shows that generative AI has immense potential in the future, and continuous monitoring of new technologies is necessary to ensure that patients receive the best possible care.
