# Supplementary material for: Applications of generative artificial intelligence in outcome prediction in intensive care medicine—a scoping review
Source: Front Digit Health. 2025 Aug 5;7:1633458. doi: 10.3389/fdgth.2025.1633458 (PMC12361121; doi:10.3389/fdgth.2025.1633458)
Supplement: Supplementary file 1 [file Datasheet1.pdf]

Supplementary Material

Table of Contents

SUPPLEMENTAL FILE 1. SEARCH STRATEGY 2

SUPPLEMENTAL FILE 2. PAPERS EXCLUDED AFTER ABSTRACT SCREENING AND, WHERE NECESSARY, FULL TEXT REVIEW 3

Supplemental File 1. Search strategy

Table S1: Search strategy

Database	Search string
IEEE Explore	("All Metadata":generative artificial intelligence OR "All Metadata":generative AI OR "All Metadata":GenAI OR "All Metadata":generative machine learning OR "All Metadata":generative adversarial network OR "All Metadata":large language model OR "All Metadata":LLM OR "All Metadata":variational autoencoder OR "All Metadata":diffucion model) AND ("All Metadata":intensive care OR "All Metadata":intensive medicine OR "All Metadata":ICU OR "All Metadata":critical care) AND ("All Metadata":outcome OR "All Metadata":sepsis OR "All Metadata":mortality OR "All Metadata":survival)
PubMed	(("generative artificial intelligence" OR "generative AI" OR genAI OR "generative machine learning" OR "generative adversarial network" OR "large language model" OR LLM OR "variational autoencoder" OR "diffusion model") AND ("intensive care"[Title/Abstract] OR "intensive medicine"[Title/Abstract] OR ICU[Title/Abstract] OR “critical care”[Title/Abstract])) AND (outcome[Title/Abstract] OR sepsis[Title/Abstract] OR mortality[Title/Abstract] OR survival[Title/Abstract])
CINAHL	(generative artificial intelligence or generative AI or generative machine learning or generative adversarial network or large language model or LLM or variational autoencoder) AND AB (intensive care or intensive medicine or ICU or critical care) AND AB (outcome or sepsis or mortality or survival)
Google Scholar	The general term "generative artificial intelligence" was used in combination with different specific keywords, including “critical”, “care”, “intensive”, “medicine”, and “ICU”:<br>allintitle: critical "generative artificial intelligence"<br>allintitle: care "generative artificial intelligence"<br>allintitle: intensive "generative artificial intelligence"<br>allintitle: medicine "generative artificial intelligence"<br>allintitle: icu "generative artificial intelligence"<br>The search was repeated three times with "generative adversarial network", “large language model”, and “variational autoencoder” replacing the term "generative artificial intelligence".
arXiv	AND all=generative artificial intelligence; AND all=intensive; AND all=medicine; AND all=outcome<br>The search was repeated three times with "generative adversarial network", “large language model”, and “variational autoencoder” replacing the term "generative artificial intelligence".

Supplemental File 2. Papers excluded after abstract screening and, where necessary, full text review

Table S2: Papers excluded after the screening of abstracts and reports where necessary

Authors	Title	Publication year	DOI	Reason for exclusion
IEEE				
A. Rozo; V. Miskovic; T. Rose; E. Keersebilck; C. Iorio; C. Varon	A Deep Learning Image-to-Image Translation Approach for a More Accessible Estimator of the Healing Time of Burns	2023	10.1109/TBME.2023.3267600	No: other area: burns, imaging, Laser Doppler imaging (LDI)
G. Kumar	A Doctor Assistance Tool: Personalized Healthcare Treatment Recommendations Journey from Deep Reinforcement Learning to Generative AI	2024	10.1109/DELCON64804.2024.10866814	No, other purpose: LLM generates treatment recommendations
M. Mahalakshmi; S. Bharadwaj; A. N. Bhuyan	A Real-Time Medical Report Analysis and AI-Powered Diagnosis: A Cloud-Based Solution for Improved Patient Care	2024	10.1109/ICAIT61638.2024.10690733	No: other purpose, patient reports used for diagnostics
L. Jiang; X. Yang; C. Yu; Z. Wu; Y. Wang	Advanced AI Framework for Enhanced Detection and Assessment of Abdominal Trauma: Integrating 3D Segmentation with 2D CNN and RNN Models	2024	10.1109/RAIIC61787.2024.10671250	No: no generative model, images: analysis of CT Scans
R. Kumar; R. Bhagwat; A. Kumar V	Advanced Neural Network Models for Predictive Analytics and Healthcare Management in Neurodegenerative Diseases	2023	10.1109/ICRASET59632.2023.10419967	No: other area, neurodegenerative diseases
Y. Wang; C. Li; Z. Wang	Advancing Precision Medicine: VAE Enhanced Predictions of Pancreatic Cancer Patient Survival in Local Hospital	2024	10.1109/ACCESS.2023.3348810	No: other area VAE-augmented framework in pancreatic cancer
M. Makram; A. Mohammcd	AI Applications in Medical Reporting and Diagnosis	2024	10.1109/MIUCC62295.2024.10783552	No: other area: liver disease, LLM to analyse images and clinical notes
F. Shah-Mohammadi; J. Finkelstein	AI-Powered Social Determinants of Health Extraction from Patient Records: A GPT-Based Investigation	2024	10.1109/AIMHC59811.2024.00028	No: other purpose, GPT (Generative Pretrained Transformer) model for extraction of addiction status from patient discharge summaries
A. R. Neravetla; S. Samreen; A. S. Mohammed; N. Jiwani; J. Logeshwaran	An Improved Lung Cancer Prediction Algorithm using Generative Adversarial Network in Modern Healthcare	2024	10.1109/ICICACS60521.2024.10498818	No: other area: lung cancer
Y. Ji; Z. Yu; Y. Wang	Assertion Detection in Clinical Natural Language Processing Using Large Language Models	2024	10.1109/ICHI61247.2024.00039	No: other area: assertion detection when extracting medical concepts from clinical notes using LLMs
Y. An; R. Qiu; L. Guo; X. Chen	Attention-based Multimodal Fusion with Adversarial Network for In-Hospital Mortality Prediction	2024	10.1109/BIBM62325.2024.10821815	No: no data are generated, prediction based on the most relevant features
K. M. S. Islam; A. S. Nipu; P. Madiraju; P. Deshpande	Autocompletion of Chief Complaints in the Electronic Health Records using Large Language Models	2023	10.1109/BigData59044.2023.10386778	No: other area, chief complaint, no ICU

Authors	Title	Publication year	DOI	Reason for exclusion
E. Estiri; H. Mirinejad	Bayesian Variational Autoencoders for Out-of-Distribution Detection in Physiological Modeling: A Case Study in Fluid Therapy	2024	10.1109/EMBC53108.2024.10781780	No: other area: a case study in fluid therapy
V. B. Oguluri; M. S. N. Raju; J. Vaka; V. Murukutla	Classification of Acute Lymphoblastic Leukemia Using MobileNet and EfficientNetB3	2023	10.1109/ICBDS58040.2023.10346455	No: other area, acute lymphoblastic leukaemia
A. Mendoza; S. Tume; K. Puri; S. Acosta; J. R. Cavallaro	Clinical Features and Physiological Signals Fusion Network for Mechanical Circulatory Support Need Prediction in Pediatric Cardiac Intensive Care Unit	2025	10.1109/JBHI.2024.3510217	No: no generative model
K. L. Prasanna; Y. N. Rao	Context-Aware Approaches in IoT-based Healthcare Systems using Deep Learning Techniques: A Study	2024	10.1109/ICAAIC60222.2024.10575875	No: other area, reviewed IoT-enabled medical care systems using context awareness
Shruthi K; Naveen K B	DEEP Learning Approach Using Modified Auto-Encoder for PPG Signal Compression	2023	10.1109/ICTBIG59752.2023.10456180	No: other purpose, encoder/decoder model for the heart's pumping capacity
W. Wu; Y. Lu; R. Mane; C. Guan	Deep Learning for Neuroimaging Segmentation with a Novel Data Augmentation Strategy	2020	10.1109/EMBC44109.2020.9176537	No: other area: neuo-imaging, a conditional Generative Adversarial Network (cGAN) which is followed by a supervised segmentation with a Convolutional Neural Network (CNN) of brain images
M. Mohammed; S. Atiani; R. A. A. Rub; F. F. Kharbat; M. Odeh	Deriving Data Modeling Requirements Using Topic Modelling in Cancer Care Context: The Case of the iCanViz Project	2023	10.1109/ICCNS58795.2023.10193611	No: other area: cancer incidence
A. M. Alshahrani; H. Farooq Ahmad; J. Hussain	Detecting Health Misinformation on Social Networking Sites Using Large Language Models and Deep Learning-based Natural Language Processing	2024	10.1109/FLLM63129.2024.10852503	No: other area, not related to EHR data
S. Nagoor; L. Hederman; K. Koidl; I. Martin-Loeches	Distinguishing Clinical Sentiment in Intensive Care Unit Clinical Notes	2024	10.1109/CBMS61543.2024.00049	No: other area: sentiment analysis in critical care notes using lexicon models, BERT models, and prompt models
M. M. Alqezweeni; V. I. Gorbachenko; O. K. Zenin; D. N. Gribkov; V. V. Potapov; I. Miltykh	Early Diagnoses of Chronic Heart Failure using Neural Network Classifier of Tensiometric Blood Test Results	2022	10.1109/ICDSIC56987.2022.10076007	No: other area: early diagnosis of chronic heart failure, training data generated with VAE because of insufficiency
D. Raj; J. A J; R. Rafeeque; R. T S; A. S; M. K P	Enhancing Seizure Detection Through Deep Learning-Based Wearable Devices	2024	10.1109/ICIC3S61846.2024.10603386	No: other area: automated seizure detection from wearable device data
W. Cai; Y. Li; Y. Chen; J. Lin; Z. Huang; P. Gao; T. R. Gadekallu; W. Wang; Y. Gao	Enhancing Weakly Supervised Semantic Segmentation with Multi-label Contrastive Learning and LLM Features Guidance	2024	10.1109/JBHI.2024.3450013	No: other area: histopathology, images, class-specific embedding extraction with LLM features guidance on histopathological images

Authors	Title	Publication year	DOI	Reason for exclusion
A. Zalewski; W. Long; A. E. W. Johnson; R. G. Mark; L. -w. H. Lehman	Estimating patient's health state using latent structure inferred from clinical time series and text	2017	10.1109/BHI.2017.7897302	No: no generative model: Hierarchical Dirichlet Processes to use multiple data sources including nursing notes
Y. Zhou; D. Dreizin; Y. Wang; F. Liu; W. Shen; A. L. Yuille	External Attention Assisted Multi-Phase Splenic Vascular Injury Segmentation With Limited Data	2022	10.1109/TMI.2021.3139637	No: other area, image augmentation, a novel framework for multi-phase splenic vascular injury segmentation
C. Sibunruang; J. Polpinij	Finding Clinical Knowledge from MEDLINE Abstracts by Text Summarization Technique	2018	10.23919/INCIT.2018.8584867	No: other area, summarising text from Medline
J. Wu; X. Ye; C. Mou; W. Dai	FineEHR: Refine Clinical Note Representations to Improve Mortality Prediction	2023	10.1109/ISDFS58141.2023.10131726	No: other area, improving the prediction accuracy using embeddings and metric learning
S. Singh; S. Singh; C. S. Raghuvanshi	Generating Synthetic Medical Data Using GAI	2025	10.1002/9781394280735.ch3	No: other area: Generative Computerized reasoning (GAI) in medicine in general
M. Ehghaghi; P. -Y. Zhou; W. Y. Cheng; S. Rajabi; C. -H. Kuo; E. -S. A. Lee	Interpretable Disease Prediction from Clinical Text by Leveraging Pattern Disentanglement	2023	10.1109/BHI58575.2023.10313408	No: other area, disease prediction, not ICU, processing clinical notes
L. -w. Lehman; W. Long; M. Saeed; R. Mark	Latent topic discovery of clinical concepts from hospital discharge summaries of a heterogeneous patient cohort	2014	10.1109/EMBC.2014.6943952	No: no generative model, Hierarchical Dirichlet Process (HDP) as a non-parametric topic modeling technique to automatically discover the latent “topic” structure of diseases, symptoms, and findings documented in hospital discharge summaries
H. -W. Hu; Y. -c. Lin; C. -H. Chia; E. Chuang; Y. Cheng Ru	Leveraging Large Language Models for Generating Personalized Care Recommendations in Dementia	2024	10.1109/iWEM59914.2024.10649066	No, other purpose: a GPT-4 based LLM, integrating it with a vector database using RAG to optimize care plan personalization
M. A. H. Zahid; J. Lee	Mortality prediction with self normalizing neural networks in intensive care unit patients	2018	10.1109/BHI.2018.8333410	No: SNN is not generative, use of ANN, but no new data produced, self normalizing neural network
A. Prasad; V. Asha; N. Tressa; D. Sharma; D. V; D. S	NeuroNet: A Deep Learning Framework for Stroke Analysis	2024	10.1109/IDCIoT59759.2024.10467659	No: other area, stroke prediction
F. Scotti; D. Magnanimi; V. M. Urbano; F. Pierri	Online feelings and sentiments across Italy during pandemic: investigating the influence of socio-economic and epidemiological variables	2020	10.1109/ASONAM49781.2020.9381463	No: other area, analysing tweets during the pandemic
R. C. Pereira; P. H. Abreu; P. P. Rodrigues	Partial Multiple Imputation With Variational Autoencoders: Tackling Not at Randomness in Healthcare Data	2022	10.1109/JBHI.2022.3172656	No: other area, not ICU, VAE to impute EHR data

Authors	Title	Publication year	DOI	Reason for exclusion
H. Xu; W. Wu; S. Nemati; H. Zha	Patient Flow Prediction via Discriminative Learning of Mutually-Correcting Processes	2017	10.1109/TKDE.2016.2618925	No: other area: patient flow prediction, data augmentation for patient flow modelling in class imbalance
J. Lee	Personalized mortality prediction for the critically ill using a patient similarity metric and bagging	2016	10.1109/BHI.2016.7455902	No: no generative model, classification based on similarity between patients
Y. Dai; S. Lokhandwala; W. Long; R. Mark; L. -w. H. Lehman	Phenotyping hypotensive patients in critical care using hospital discharge summaries	2017	10.1109/BHI.2017.7897290	No: no generative model, Hierarchical Dirichlet Process, discovered topic structure in phenotyping critically-ill patients who experienced hypotensive episodes
D. Hao; M. Negahdar	Predicting Outcomes in Long COVID Patients with Spatiotemporal Attention	2023	10.1109/ICHI57859.2023.00031	No: other area: Long COVID, spatial relation, a spatiotemporal attention mechanism to weigh feature importance jointly from the temporal dimension and feature space
N. S. Damanhuri; Y. S. Chiew; P. Docherty; P. Geoghegan; G. Chase	Respiratory airway resistance monitoring in mechanically ventilated patients	2012	10.1109/IECBES.2012.6498135	No: other purpose, assesses the efficacy of a physically derived airway branching model (ABM) to capture clinical data
J. Lenin; A. Komathi; H. Vijayan; A. R. Rathinam; A. Kasthuri; C. Srinivasan	Revolutionizing Healthcare With Cloud Computing: The Impact of Clinical Decision Support Algorithm	2023	10.1109/ICAIIHI57871.2023.10489022	No: other area: advantages of cloud computing
E. Estiri; H. Mirinejad	Robust Nonlinear State Space Model Identification for Hemorrhage Resuscitation	2023	10.1109/BHI58575.2023.10313391	No: other area, predicting hemodynamic responses in hemorrhage resuscitation
N. M. Matasyoh; F. Mathis-Ullrich; R. A. Zeineldin	SAMSurg: Surgical Instrument Segmentation in Robotic Surgeries Using Vision Foundation Model	2024	10.1109/ACCESS.2024.3520386	No: other area, Integrating Minimally Invasive Surgery, instrument segmentation
D. Restrepo; L. F. Nakayama; R. G. Dychiao; C. Wu; L. G. McCoy; J. C. Artiaga; M. Cobanaj; J. Matos; J. Gallifant; D. S. Bitterman; V. Ferrer; Y. Aphinyanaphongs; L. Anthony Celi	Seeing Beyond Borders: Evaluating LLMs in Multilingual Ophthalmological Question Answering	2024	10.1109/ICHI61247.2024.00089	No: other area: ophthalmology
P. Bookanakere; S. S; S. Saniya; A. R; S. K. S	Textual Therapies: Harnessing Language Models for Mental Health Insights	2024	10.1109/ACROSET62108.2024.10743711	No: other area, OpenAI's Generative Pre-Trained Transformer 2 (GPT-2) model to predict the presence of a mental illness
M. Poongodi; M. Malviya; M. Hamdi; H. T. Rauf; S. Kadry; O. Thinnukool	The Recent Technologies to Curb the Second-Wave of COVID-19 Pandemic	2021	10.1109/ACCESS.2021.3094400	No: other area, out of scope, general issues of pandemic

Authors	Title	Publication year	DOI	Reason for exclusion
M. Sharma; B. Bhagat; V. Kumari; Bhumi; N. Sharma; D. Singh	A Comparative Analysis of CNN and Transfer Learning Algorithms in Predicting Alzheimer's Disease Stages Using Neuroimaging	2024	10.1109/OTCON60325.2024.10688232	No: other area: Alzheimer's Disease
B. Cánovas-Segura; F. Zerbato; B. Oliboni; C. Combi; M. Campos; A. Morales; J. M. Juarez; R. Marin; F. Palacios	A Process-Oriented Approach for Supporting Clinical Decisions for Infection Management	2017	10.1109/ICHI.2017.73	No: other area: process for supporting clinical decisions for infection management
K. H. Khalaf; A. M. Khiavi; D. H. Abd	Adversarial Ensemble Learning for Mortality Prediction in Intensive Care Units	2024	10.1109/DeSE63988.2024.10912041	No: no generative model
F. Zhao; J. Nie; M. Ma; X. Chen; X. He; B. Wang; Y. Hou	Assessing the Role of Different Heterogeneous Regions in DCE-MRI for Predicting Molecular Subtypes of Breast Cancer based on Network Architecture Search and Vision Transformer	2023	10.1109/EMBC40787.2023.10340066	No: other area: breast cancer
M. Bazargani; M. J. Heidari; M. Anvari-Fard; H. Soltanian-Zadeh	Comparison of Six MRI Sequence Combinations for Brain Tumor Segmentation Using U-Net	2024	10.1109/ICBME64381.2024.10895163	No: other area: brain tumor segmentation
M. Shehata; F. Khalifa; A. Soliman; M. Ghazal; F. Taher; M. A. El-Ghar; A. C. Dwyer; G. Gimel'farb; R. S. Keynton; A. El-Baz	Computer-Aided Diagnostic System for Early Detection of Acute Renal Transplant Rejection Using Diffusion-Weighted MRI	2019	10.1109/TBME.2018.2849987	No: other area: acute renal transplant rejection
Z. Ishak; A. S. A. Amran; M. D. Esfahani; R. K. Ayyasamy; M. O. B. Abdullah; Y. J. Ann	Determining the Readiness Factors to Adapt Blockchain Technology in the Palm Oil Industry to Facilitate Circular Economy Transition	2024	10.1109/AiDAS63860.2024.10730330	No: other area: blockchain technology in the palm oil industry
Y. Liu; X. Wang; X. Wang; Z. Wang; J. Kurths	Diffusion Source Inference for Large-Scale Complex Networks Based on Network Percolation	2025	10.1109/TNNLS.2023.3321767	No: other area: diffusion source inference
P. Busaranuvong; E. Agu; D. Kumar; S. Gautam; R. S. Fard; B. Tulu; D. Strong	Guided Conditional Diffusion Classifier (ConDiff) for Enhanced Prediction of Infection in Diabetic Foot Ulcers	2025	10.1109/OJEMB.2024.3453060	No: other area: prediction of infection in diabetic foot ulcers
J. Huang	Notice of Violation of IEEE Publication Principles: Computational Model of Protein-Bound Uremic Toxins Kinetics During Hemodialysis and Dose Evaluation of Accurate Dialysis Using Deep Learning Techniques	2020	10.1109/ACCESS.2020.2990725	No: other area: model of protein-bound uremic toxins kinetics
D. Vrba; L. Malena; J. Albrecht; J. Fricova; M. Anders; R. Rokyta; D. Rodrigues; J. Vrba	Numerical Analysis of Transcranial Magnetic Stimulation Application in Patients With Orofacial Pain	2022	10.1109/TNSRE.2022.3156703	No: other area: transcranial magnetic stimulation
C. Kim; C. U. Lehmann; D. Hatch; J. S. Schildcrout; D. J. France; Y. Chen	Provider Networks in the Neonatal Intensive Care Unit Associate with Length of Stay	2019	10.1109/CIC48465.2019.00024	No: other area: provider networks, length of stay
J. S. Preston; T. Tasdizen; C. M. Terry; A. K. Cheung	Using the Stochastic Collocation Method for the Uncertainty Quantification of Drug Concentration Due to Depot Shape Variability	2009	10.1109/TBME.2008.2009882	No: other area: drug concentration
Q. Wu; W. Huang; S. Wang; H. Yu; L. Wang;	A generative adversarial network-based CT image standardization model	2021	10.1109/EMBC46164.2021.9630092	No: other area: lung cancer, generative

Authors	Title	Publication year	DOI	Reason for exclusion
Z. Wu; Y. Zhu; Z. Liu; H. Ma; J. Tian	for predicting progression-free survival of lung cancer			adversarial network (GAN)-based model to convert heterogenous CT images into standardized CT images with uniform slice thickness and reconstruction kernel to increase the generalization of the predictive model
M. Noaeen; S. Amini; S. Bhasker; Z. Ghezelsefli; A. Ahmed; O. Jafarinezhad; Z. S. H. Abad	Unlocking the Power of EHRs: Harnessing Unstructured Data for Machine Learning-based Outcome Predictions	2023	10.1109/EMBC40787.2023.10340232	No: no generative model
**Pubmed**				
Subudhi S, Verma A, Patel AB, Hardin CC, Khandekar MJ, Lee H, McEvoy D, Stylianopoulos T, Munn LL, Dutta S, Jain RK.	Comparing machine learning algorithms for predicting ICU admission and mortality in COVID-19	2021	10.1038/s41746-021-00456-x	No: no generative model
Li Y, Song S, Zhu L, Zhang X, Mou Y, Lei M, Wang W, Tao Z.	Machine learning-based prediction model for patients with recurrent Staphylococcus aureus bacteremia	2025	10.1186/s12911-025-02878-z	No: no generative model
Barrios EL, Mazer MB, McGonagill PW, Bergmann CB, Goodman MD, Gould RW, Rao M, Polcz VE, Davis RJ, Del Toro DE, Dirain ML, Dram A, Hale LO, Heidarian M, Kim CY, Kucaba TA, Lanz JP, McCray AE, Meszaros S, Miles S, Nelson CR, Rocha IL, Silva EE, Ungaro RF, Walton AH, Xu J, Zeumer-Spataro L, Drewry AM, Liang M, Bible LE, Loftus TJ, Turnbull IR, Efron PA, Remy KE, Brakenridge SC, Badovinac VP, Griffith TS, Moldawer LL, Hotchkiss RS, Caldwell CC.	Adverse outcomes and an immunosuppressed endotype in septic patients with reduced IFN-γ ELISpot	2024	10.1172/jci.insight.175785	No: no generative model
Mateo J, Payen D, Ghout I, Vallée F, Lescot T, Welschbillig S, Tazarourte K, Azouvi P, Weiss JJ, Aegerter P, Vigué B.	Impact of extended monitoring-guided intensive care on outcome after severe traumatic brain injury: A prospective multicentre cohort study (PariS-TBI study)	2017	10.1080/02699052.2017.1370554	No: no generative model
Brakenridge SC, Efron PA, Stortz JA, Ozrazgat-Baslanti T, Ghita G, Wang Z, Bihorac A, Mohr AM, Brumback BA, Moldawer LL, Moore FA.	The impact of age on the innate immune response and outcomes after severe sepsis/septic shock in trauma and surgical intensive care unit patients	2018	10.1097/TA.0000000000001921	No: no generative model
Stortz JA, Mira JC, Raymond SL, Loftus TJ,	Benchmarking clinical outcomes and the immunocatabolic phenotype of	2018	10.1097/TA.0000000000001758	No: no generative model

Authors	Title	Publication year	DOI	Reason for exclusion
Ozrazgat-Baslanti T, Wang Z, Ghita GL, Leeuwenburgh C, Segal MS, Bihorac A, Brumback BA, Mohr AM, Efron PA, Moldawer LL, Moore FA, Brakenridge SC.	chronic critical illness after sepsis in surgical intensive care unit patients			
Kuo RN, Chen W, Shau WY.	Risk factors for disease progression and clinical outcomes in patients with COVID-19 in Taiwan: a nationwide population-based cohort study	2025	10.1186/s12890-024-03468-x	No: no generative model
Subudhi S, Voutouri C, Hardin CC, Nikmaneshi MR, Patel AB, Verma A, Khandekar MJ, Dutta S, Stylianopoulos T, Jain RK, Munn LL.	Strategies to minimize heterogeneity and optimize clinical trials in Acute Respiratory Distress Syndrome (ARDS): Insights from mathematical modelling	2022	10.1016/j.ebiom.2021.103809	No: no generative model
Efron PA, Brakenridge SC, Mohr AM, Barrios EL, Polcz VE, Anton S, Ozrazgat-Baslanti T, Bihorac A, Guirgis F, Loftus TJ, Rosenthal M, Leeuwenburgh C, Mankowski R, Moldawer LL, Moore FA.	The persistent inflammation, immunosuppression, and catabolism syndrome 10 years later	2023	10.1097/TA.0000000000004087	No: other purpose: persistent inflammation, immunosuppression, and catabolism syndrome
Joe B, Park Y, Hamm J, Shin I, Lee J.	Exploiting Missing Value Patterns for a Backdoor Attack on Machine Learning Models of Electronic Health Records: Development and Validation Study	2022	10.2196/38440	No: other purpose: missing value patterns for a backdoor attack
El Gharib K, Jundi B, Furfaro D, Abdulnour RE.	AI-assisted human clinical reasoning in the ICU: beyond "to err is human"	2024	10.3389/frai.2024.1506676	No: other purpose: AI-assisted human clinical reasoning
Kuo NI, Polizzotto MN, Finfer S, Garcia F, Sönnerborg A, Zazzi M, Böhm M, Kaiser R, Jorm L, Barbieri S.	The Health Gym: synthetic health-related datasets for the development of reinforcement learning algorithms	2022	10.1038/s41597-022-01784-7	No: other purpose: synthetic data for reinforcement learning
Qin W, Wang J, Huang F, Chen X.	Management for lower limb malperfusion in acute type a aortic dissection patients	2023	10.1177/02676591221116491	No: lower limb malperfusion (LLM)
van Nispen C, Long B, Koyfman A.	High risk and low prevalence diseases: Stevens Johnson syndrome and toxic epidermal necrolysis	2024	10.1016/j.ajem.2024.04.001	No: other purpose: Stevens Johnson syndrome and toxic epidermal necrolysis
Huespe IA, Echeverri J, Khalid A, Carboni Bisso I, Musso CG, Surani S, Bansal V, Kashyap R.	Clinical Research With Large Language Models Generated Writing-Clinical Research with AI-assisted Writing (CRAW) Study	2023	10.1097/CCE.0000000000000975	No: other purpose: AI-assisted writing
Yang H, Hu M, Most A, Hawkins WA, Murray B, Smith SE, Li S, Sikora A.	Evaluating accuracy and reproducibility of large language model performance on critical care assessments in pharmacy education	2025	10.3389/frai.2024.1514896	No: other purpose: pharmacy education
Doshi R, Amin KS, Khosla P, Bajaj SS, Chheang S, Forman HP.	Quantitative Evaluation of Large Language Models to Streamline Radiology Report Impressions: A Multimodal Retrospective Analysis	2024	10.1148/radiol.231593	No: other area: simplify radiology report impressions
CINAHL				

Authors	Title	Publication year	DOI	Reason for exclusion
Baum, Aaron ; Batniji, Rajaie ; Ratcliffe, Hannah ; DeGosztonyi, Margalit ; Basu, Sanjay	Supporting Rising-Risk Medicaid Patients Through Early Intervention	20250201	10.3390/nursrep15020055	No: other area: supporting rising-risk Medicaid patients
Alderden, Jenny ; Johnny, Jace ; Brooks, Katie R. ; Wilson, Andrew ; Yap, Tracey L. ; Zhao, Yunchuan ; van der Laan, Mark ; Kennerly, Susan	Explainable Artificial Intelligence for Early Prediction of Pressure Injury Risk.	20241101	10.1056/CAT.24.0060	No: no generative model used for outcome prediction, but synthetic data used for the dashboard, Hospital-acquired pressure injuries, synthetic data
Gondode, Prakash G. ; Khanna, Puneet ; Sharma, Pradeep ; Duggal, Sakshi ; Garg, Neha	End-of-life Care Patient Information Leaflets--A Comparative Evaluation of Artificial Intelligence-generated Content for Readability, Sentiment, Accuracy, Completeness, and Suitability: ChatGPT vs Google Gemini.	20240901	10.4037/ajcc2024856	No: artificial intelligence (AI) in addressing the informational gap by analyzing patient information leaflets
Decker, Hannah ; Trang, Karen ; Ramirez, Joel ; Colley, Alexis ; Pierce, Logan ; Coleman, Melissa ; Bongiovanni, Tasce ; Melton, Genevieve B. ; Wick, Elizabeth	Large Language Model–Based Chatbot vs Surgeon-Generated Informed Consent Documentation for Common Procedures.	20231001	10.1177/02676591221116491	No: LLM-based chatbot generated more readable, complete, and accurate consent documentation than the surgeons
Mateo, Joaquim ; Payen, Didier ; Ghout, Idir ; Vallée, Fabrice ; Lescot, Thomas ; Welschbillig, Stephane ; Tazarourte, Karim ; Azouvi, Philippe ; Weiss, Jean-Jacques ; Aegerter, Philippe ; Vigué, Bernard	Impact of extended monitoring-guided intensive care on outcome after severe traumatic brain injury: A prospective multicentre cohort study (PariS-TBI study).	20231009	10.1001/jamanetworkopen.2023.36997	No: low level of monitoring (LLM)
Gillies, Christopher E. ; Taylor, Daniel F. ; Cummings, Brandon C. ; Ansari, Sardar ; Islim, Fadi ; Kronick, Steven L. ; Medlin, Richard P. ; Ward, Kevin R. ; Medlin, Richard P Jr	Demonstrating the consequences of learning missingness patterns in early warning systems for preventative health care: A novel simulation and solution.	20171101	10.1080/02699052.2017.1370554	No: early warning systems for preventative health care
Glass, Hannah C. ; Wood, Thomas R. ; Comstock, Bryan A. ; Numis, Adam L. ; Bonifacio, Sonia L. ; Cornet, Marie-Coralie ; Gonzalez, Fernando F. ; Morell, Adriana ; Kolnik, Sarah E. ; Li, Yi ; Mathur, Amit ; Mietzsch, Ulrike ; Wu, Tai-Wei ; Wusthoff, Courtney J. ; Thoresen, Marianne ; Heagerty, Patrick J. ; Juul, Sandra E. ; Wu, Yvonne W.	Predictors of Death or Severe Impairment in Neonates With Hypoxic-Ischemic Encephalopathy.	20201001	10.1016/j.jbi.2020.103528	No: no generative model
Khubchandani, Jasmine A. ; Ingraham, Angela M. ; Daniel, Vijaya T. ; Ayturk, Didem ; Kiefe,	Geographic Diffusion and Implementation of Acute Care Surgery: An Uneven Solution to the	20241205	10.1001/jamanetworkopen.2024.49188	No: other area

Authors	Title	Publication year	DOI	Reason for exclusion
Catarina I. ; Santry, Heena P.	National Emergency General Surgery Crisis.			
Sarton, Benjamine ; Jaquet, Pierre ; Belkacemi, Djida ; de Montmollin, Etienne ; Bonneville, Fabrice ; Sazio, Charline ; Frérou, Aurelien ; Conrad, Marie ; Daubin, Delphine ; Chabanne, Russell ; Argaud, Laurent ; Dailler, Frédéric ; Brulé, Noëlle ; Lerolle, Nicolas ; Maestraggi, Quentin ; Marechal, Julien ; Bailly, Pierre ; Razazi, Keyvan ; Mateos, Francois ; Guidet, Bertrand	Assessment of Magnetic Resonance Imaging Changes and Functional Outcomes Among Adults With Severe Herpes Simplex Encephalitis.	20180201	10.1001/jamasurg.2017.3799	No: no generative model
Berghöfer, Anne ; Afraz, Farideh C. ; Dreher, Carsten	Regional global budgets to promote integrated mental health care in Germany...22nd International Conference on Integrated Care, May 23-25, 2022, Odense, Denmark.	20210727	10.1001/jamanetworkopen.2021.14328	No: other area
Taylor-Piliae RE	Establishing evidence-based practice: issues and implications in critical care nursing.	20221002	10.5334/ijic.ICIC22194	No: no generative model
Google scholar				
Hajijama, Sameera; Juneja, Deven; Nasa, Prashant;	Large Language Model in Critical Care Medicine: Opportunities and Challenges	2024	10.5005/jp-journals-10071-24743	No study, comment
Hwai, Haw; Ho, Yi-Ju; Wang, Chih-Hung; Huang, Chien-Hua;	Large language model application in emergency medicine and critical care	2024	10.1016/j.jfma.2024.08.032	No study, comment
Fehr, Joël;	LLaMA-Care A Multimodal Medical Large Language Model for Hospital Discharge Instruction Generation	2024	10.48550/arXiv.2406.02350	No: other purpose: discharge instruction generation
arXiv				
Braja Gopal Patra, Lauren A. Lepow, Praneet Kasi Reddy Jagadeesh Kumar, Veer Vekaria, Mohit Manoj Sharma, Prakash Adekkanattu, Brian Fennessy, Gavin Hynes, Isotta Landi, Jorge A. Sanchez-Ruiz, Euijung Ryu, Joanna M. Biernacka, Girish N. Nadkarni, Ardesheer Talati, Myrna Weissman, Mark Olfson, J. John Mann, Alexander W. Charney, Jyotishman Pathak	Extracting Social Support and Social Isolation Information from Clinical Psychiatry Notes: Comparing a Rule-based NLP System and a Large Language Model	2024	10.1093/jamia/ocae260	No: other area: Psychiatry
Angela Zhang, Mert Yuksekgonul, Joshua Guild, James Zou, Joseph C. Wu	ChatGPT Exhibits Gender and Racial Biases in Acute Coronary Syndrome Management	2023	10.48550/arXiv.2311.14703	No: other area: gender and racial biases of ChatGPT
